# How QOF is shaping primary care review consultations: a longitudinal qualitative study

**DOI:** 10.1186/1471-2296-14-103

**Published:** 2013-07-21

**Authors:** Carolyn A Chew-Graham, Cheryl Hunter, Susanne Langer, Alexandra Stenhoff, Jessica Drinkwater, Elspeth A Guthrie, Peter Salmon

**Affiliations:** 1 General Practice Research, Research Institute, Primary Care and Health Sciences, Keele University, Keele, Staffordshire ST5 5BG, England; 2 Institute of Population Health, Primary Care Centre, and National School for Primary Care Research, University of Manchester, 5th floor, Williamson Building Oxford Road, Manchester M13 9PL, England; 3 Health Services Research Unit, Nuffield Department of Population Health, University of Oxford, Rosemary Rue Building, Old Road Campus, Oxford OX3 7LF, England; 4 Mental Health and Well-Being, Department of Mental and Behavioural Health Sciences, University of Liverpool, Liverpool L69 3GB, England; 5 Department of Mental and Behavioural Health Sciences, Institute of Psychology Health & Society, University of Liverpool, Whelan Building, Liverpool L69 3GB, England; 6 Psychological Medicine & Medical Psychotherapy, Manchester Royal Infirmary, Rawnsley Building, Manchester M13 9WL, England; 7 Division of Clinical Psychology, Institute of Psychology, Health and Society, University of Liverpool, Liverpool L69 3GB, England

**Keywords:** Primary care, Long-term conditions, Quality and Outcomes Framework (QOF), Consultations, Longitudinal qualitative research

## Abstract

**Background:**

Long-term conditions (LTCs) are increasingly important determinants of quality of life and healthcare costs in populations worldwide. The Chronic Care Model and the NHS and Social Care Long Term Conditions Model highlight the use of consultations where patients are invited to attend a consultation with a primary care clinician (practice nurse or GP) to complete a review of the management of the LTC. We report a qualitative study in which we focus on the ways in which QOF (Quality and Outcomes Framework) shapes routine review consultations, and highlight the tensions exposed between patient-centred consulting and QOF-informed LTC management.

**Methods:**

A longitudinal qualitative study. We audio-recorded consultations of primary care practitioners with patients with LTCs. We then interviewed both patients and practitioners using tape-assisted recall. Patient participants were followed for three months during which the research team made weekly contact and invited them to complete weekly logs about their health service use. A second interview at three months was conducted with patients. Analysis of the data sets used an integrative framework approach.

**Results:**

Practitioners view consultations as a means of ‘surveillance’ of patients. Patients present themselves, often passively, to the practitioner for scrutiny, but leave the consultation with unmet biomedical, informational and emotional needs. Patients perceived review consultations as insignificant and irrelevant to the daily management of their LTC and future healthcare needs. Two *deviant* cases, where the requirements of the ‘review’ were subsumed to meet the patient’s needs, focused on cancer and bereavement.

**Conclusions:**

Routine review consultations in primary care focus on the biomedical agenda set by QOF where the practitioner is the expert, and the patient agenda unheard. Review consultations shape patients’ expectations of future care and socialize patients into becoming passive subjects of ‘surveillance’. Patient needs outside the narrow protocol of the review are made invisible by the process of review except in extreme cases such as anticipating death and bereavement. We suggest how these constraints might be overcome.

## Background

Long-term conditions (LTCs) are increasingly important determinants of quality of life and healthcare costs in populations worldwide [1]. Primary care is seen as the optimal context to deliver care for people with long-term conditions because it is accessible, efficient, and can tackle inequalities related to socioeconomic deprivation [2,3]. There are major initiatives ongoing to improve quality of care and achieve better health in these populations [4,5]. The Chronic Care Model (http://www.improvingchroniccare.org) and the NHS and Social Care Long Term Conditions Model (http://www.dh.gov.uk) highlight key system components which need to be addressed in order to deliver effective care for people with LTCs [5,6], including delivery system design, decision support, clinical information systems and self-management support. In the United Kingdom, the Quality and Outcomes Framework (QOF) incentivizes professional activity, financially rewarding the practice when evidence-based care in line with clinical quality indicators has been demonstrated [7]. Changes in system design to operationalize QOF include the use of pre-booked consultations between an invited patient and a primary care clinician (practice nurse or GP) to complete a review of the patient and their LTC. In addition, clinical information systems (computer templates composed of ‘tick-boxes’) have been developed to help professionals deliver care in line with these QOF indicators [7], as well as providing a record to ensure payment to the practice against the quality indicators. Doran and colleagues report that improvements associated with financial incentives seem to have been achieved at the expense of small detrimental effects on aspects of care that were not incentivized, suggesting that practices divert attention from non-incentivized activities to those associated with achieving QOF points [8]. It is suggested that the use of computer templates may reinforce a checklist approach to the consultation and reduce opportunities for self-management dialogue [9]. Pay-for-performance systems operating in other health system [10] lead to clinicians doing the work they are paid for, and no other work [11].

The consultation has been described as the cornerstone of general practice: *“The essential unit of medical practice is the occasion when, in the intimacy of the consulting room or sick room, a person who is ill, or believes himself to be ill, seeks the advice of a doctor whom he trusts. This is the consultation and all else in the practice of medicine follows from it.”*[12] Balint [13] described the intrinsic therapeutic value of the doctor-patient relationship which came to assume an importance that perhaps outweighed technical aspects of care [14]. The management of patients with LTCs now takes place within clinician-initiated, time-constrained consultations.

Within the consultation literature, for both general practitioners (GPs) and practice nurses (PNs), there is an emphasis on *personalized care*, whereby the clinician takes account of an individual’s full range of needs, including health, personal, social, economic, educational, mental health, ethnic and cultural background, which can impact on a person’s total health and well-being [15]. Linked to this is the concept of ‘patient-centredness’: adopting a broader, biopsychosocial perspective of illness, taking account of the ‘patient as a person’ and understanding their unique experience of illness [16]. The Royal College of Nursing describe the key concepts of person-centred care as respect and holism, power and empowerment, choice and autonomy and empathy and compassion [17]. Stewart proposed that a useful way of understanding the concept of patient-centeredness may be to consider what it is not: not technology-centered, not doctor-centered, not hospital-centered or disease-centered [18]. Patient-centred decision making is associated with improved health care outcomes [19].

This paper reports the results of a nested longitudinal qualitative study within the CHOICE (Choosing Health Options In Chronic care Emergencies) research programme [20], focused on patients with one or more of the following conditions: asthma, diabetes, CHD or COPD, which aimed to explore the role of the consultation in shaping patients’ behaviours and future use of health services. In this paper we focus on the ways in which QOF shapes routine review consultations, and highlight the tensions exposed between operating in a patient-centred manner and QOF-informed LTC management.

## Methods

Ethics approval from North West 8 Research Committee, Greater Manchester East 10/H1013/74.

This was a longitudinal qualitative study of primary care review consultations with patients with LTCs. Combining interview data from multiple perspectives improves understanding of relationships, perceptions, and needs between patients and professionals over time [21]. The advantage is that what was said in the consultation can be seen and compared with what was intended by the health professional and heard by the patient.

### Data collection

Primary care consultations with patients with one of asthma, COPD, CHD, or diabetes, were audio-recorded with both patients’ and practitioners’ consent. General practitioners (GPs) and practice nurses (PNs) were interviewed, with consent, usually within two weeks after the recorded consultation and these interviews used the method of tape-assisted recall (TAR) [22]. TAR is an established method that has been used both in primary [22] and secondary care settings [23,24]. Playing back sections of the recording facilitated respondent recall and helped to anchor their reflections in specific consultations. Patients were interviewed, with consent, at baseline and after 12 weeks, again using TAR. The researchers maintained regular telephone contact with patients during follow-up in order to discuss decision-making about changes in symptoms and help-seeking. Repeated patient interviews and regular contact generate a richer understanding of needs and experiences [25].

### Recruitment

GP practices, in one Primary Care Trust in North West England, were invited to participate in the study, sampling for maximum geographical spread and diversity of prevalence rates across GP practices and conditions. There were two methods of identifying relevant consultations within the different practices: either the researchers attended a practice for a specified chronic disease clinic and recruited waiting patients; or the GP or PN identified patients on their appointment list with asthma, diabetes, CHD or COPD and indicated to reception staff which patients could be approached by the researcher. Patients were handed an information sheet by reception staff and invited to speak to the researcher. If a patient expressed interest, the researcher discussed the study and the participant was asked to consent to their consultation being audio-recorded. After the consultation, patient contact details were taken, consent affirmed, and a date for initial interview arranged.

Sampling was purposive for maximum variation across conditions (asthma, diabetes, COPD, CHD), health care practitioners (GP or PN), and for patients across gender and ethnicity. The end of recruitment was determined when analytical saturation had been achieved and no new themes were identified in the data set.

### Analysis

Data were anonymised and transcribed verbatim. Analysis was inductive and took a constant comparative approach, combining cross-case and within-case analysis [24]. The researchers familiarized themselves with the audio-recordings and transcripts, and discussion amongst the team identified themes documented in a set of continually updated analysis notes. Different data sources (consultation, healthcare practitioner (HCP) interview, Patient baseline interview, Patient follow-up interview) were synthesized into the cases presented here. We paid particular attention to deviant cases that modified our initial analysis.

## Results

Of 39 general practices invited to take part, 6 practices agreed to participate. Sixty five patients were approached of whom 34 were recruited for this study. We generated 18 cases with a complete set of recordings (consultation, patient baseline interview, patient follow-up interview, HCP interview) and a further 16 cases with a partial set of recordings. The primary reasons for missing data were: practitioners failing to record a consultation (n = 5), patients deciding not to take part in an interview but giving permission for consultation to be kept (n = 7), and patients dropping out or being excluded from follow-up due to health concerns (n = 5). A total of 88 transcripts were analyzed for this study. Tables describing number of respondents and recordings, and respondent characteristics are included (Additional file 1).

In this paper, we present the consultation as the starting point of the analysis. In 27 consultations, we identified divergence between patient presentation and HCP response, and these points were explored in the TAR interviews where possible. We label these points of divergence as patients’ informational, emotional or biomedical needs not being met. We present two ‘deviant cases’; one where the GP actively shifts the direction of the consultation to the patient’s concerns, and a second where the GP uses the consultation to support the patient following bereavement.

Data are presented as extracts from the consultations, with data from the linked interviews. Data are annotated with study identifiers. The ellipsis in parentheses (…) signifies omitted text. Square brackets denote explanatory text.

In the first case, there was a missed opportunity for the PN to explore the patient’s knowledge and understanding of COPD, its management, and the place of nebulizers in the consultation. The PN, by not exploring the patient’s knowledge and concerns, and not conveying her thinking about the possible use of nebulizers, caused confusion for the patient, and left him with a sense that nothing can be done to help him. He reports despondency and disillusionment with his care in both interviews. He is socialized into attending the review but has limited expectations of what it will achieve (Additional file 2).

In the second cases, the patient, in the midst of a divorce, recognizes in the consultation that the management of her asthma has been impaired by recent stressors. The PN acknowledges stress as one of the “*triggers*” for the patient’s asthma, but does not empathize with the patient in the consultation or explore the issue beyond this acknowledgement. Instead, the PN seems to use the patient’s fear of further exacerbations to encourage compliance with medication and the review process itself. In the interview, the PN does reflect on the stress the patient is under, but interprets the patient’s apparent poor compliance as due to the patient’s inherent characteristics (‘anxious lady’) and prioritizing her concerns over her symptoms and management. The patient suggests that the PN’s agenda of ensuring compliance took prominence over the patient’s own explanations and concerns. As a result of this brutal socialisation into what can and cannot be talked about in routine reviews, the patient’s expectations of the reviews are changed by the time of follow-up, when she describes the limits of her relationship with the PN (Additional file 3).

In the third case, the patient’s presentation indicates possible memory problems, which the GP does not address whilst conducting the review, instead bypassing them to focus on the QOF template of blood and urine tests. Although the GP seemed aware that the patient was having difficulty understanding how to organize blood tests, he dealt with this confusion by directing the patient to the reception staff, rather than explore possible memory problems. The patient and his wife seem superficially engaged in the process of review, but later describe feeling that the review had added nothing to his care, which remains fragmented, with problems unaddressed. Fragmentation is observable in other aspects of the patient’s care beyond the review, despite the practitioner arguing for the review’s essential role in providing “holistic” care (Additional file 4).

### Deviant cases

There were, however, two *deviant cases* where the requirements of the ‘review’ were subsumed to meet the patient’s needs.

In the first deviant case, he GP initially follows QOF and mentions the ‘borderline’ diabetes control but soon shifts the emphasis to the patient’s concern about her cancer–related issues: pain control, information (side-effects of medication), advocacy (liaising with specialists over results of tests). This recognition of the patient’s real concerns strengthened the relationship between the patient and GP, and the patient was grateful that the GP agreed to find out about her blood tests and offer further follow-up (Additional file 5).

In the second deviant case the GP-initiated consultation begins off as a routine medication review but the GP swiftly moves to explore the effects of patient’s recent bereavement, enabling the patient to talk openly with the GP about his feelings, which he reported that he valued. The GP views the review as an ideal opportunity to ensure that the patient has support at this time. The GP deliberately uses the procedure of routine reviews to pro-actively follow up and engage with this patient in a holistic manner (Additional file 6).

## Discussion

### Summary

Almost all the consultation recordings identified opportunities for GPs and PNs within the review consultations to respond to patient needs and broaden the agenda. Patient cues and concerns brought up during the review process were often missed or disregarded by practitioners. These concerns included biomedical problems that patients presented, unrelated to the LTC under review, and emotional and informational needs which would affect ongoing management of the LTCs. Appointments seem to be focused on delivering QOF, and the procedures relating to QOF dictated the talk and actions of HCPs and shaped the consultation. Patient priorities and concerns were made invisible or unexplored as a result of the dominance of QOF. Over time, patients perceived these review consultations as irrelevant to the daily management of their LTC, becoming socialized into reduced expectations for the routine care offered by HCPs.

However, there were two *deviant* cases where the GP responded to the patient’s cues, or where the GP actively shifted the focus of the consultation. In our study it was only GPs, not practice nurses, who demonstrated a willingness to go ‘off-QOF’ within routine reviews. It may be that the GP has the authority within primary care to be flexible and go ‘off-QOF’ and Practice nurses may be under more pressure (as employees of the GPs) to conform to QOF requirements. It may be that there was something unique about these consultations which changed the context of the review sufficiently to allow the GP to respond to patient cues. In both cases, these were ‘special’ patients where the focus of the consultation was re-oriented to be about symptoms related to cancer and bereavement.

#### Comparisons with previous literature

Nettleton [26] suggests that the focus on the *consultation as the centre of primary care* in the literature was due to the need of the general practice profession to find something on which to base their expertise:

“Floundering in the face of hospital medicine [GPs] searched for a distinct body of knowledge on which they could base their own professional expertise. They found their alternative to the biomedical approach in the form of biographical and holistic medicine. The biographical approach asserts the individuality of the patient, the unity of the psyche and soma and the need to get beyond the presenting symptoms to explore the history and circumstances of the patient’s life.” (p247)

However, as reported by Charles-Jones [27], GPs are no longer configuring themselves, or being configured, as concerned with people as psychosocial beings, only with them as biomedical problems (with some exceptions, such as the *mentally ill* and the *dying*). A government policy agenda that strives to apply increasingly active managerial values to the NHS encourages this retreat by GPs to a more purist biomedical space. Within this space, GPs and PNs can embrace ‘evidenced-based medicine’ (EBM Working Group 1992), and their performance is more easily measured by biomedical targets. We see in our study that discussion of cancer or distress due to bereavement were features of the consultations where the GPs were able to deviate from the bio-medical agenda, and sideline the computer template. Outside these extreme cases, the patients’ experiences of living with LTCs were disregarded. Each illness was dealt with individually according to a single QOF template. Whilst patients in consultations with nurses attempted to raise topics for discussion such as divorce, where they thought this had impacted on their LTC, the practice nurses did not respond to such cues and did not broaden the discussion. Similarly, we see consultations in which GPs did not respond to other concerns raised by patients, the GP adhering to the QOF-dictated review.

Previous studies suggest that the quality of clinical care has improved because of QOF, without detriment to the inter-personal aspects of care during consultations [28,29]. However, a survey suggested that contextual issues, including the introduction of the QOF, have contributed to difficulties in providing ‘holistic’ care [29], and Lester [11] and colleagues describe how work that is not incentivized through QOF is not prioritized in general practice. In addition Davies [30] describes the ‘crowded’ primary care consultation with competing agendas, in which QOF is an important factor, and one of the major challenges facing HCPs is how they balance these different, often equally legitimate, agendas. Stewart et al [18] suggest that a consultation cannot be patient-centred if it is disease or technology-centred – which QOF undoubtedly makes it. Previous professional accounts have attributed difficulties in providing person-centred care and in supporting self-management [16] to the presence of QOF [31] in the consultation. Our study, utilizing the rich data from a longitudinal study, including recorded consultations and interviews, adds to this literature, demonstrating how review consultations are focused around the demands of QOF, to the detriment of patients’ concerns and expectations.

### Strengths and limitations

The use of qualitative longitudinal research on the primary care consultation is rare, and no study has so far combined these approaches with a focus on patients with LTCs, whose care is a priority of health policy and primary care practice. Repeated patient interviews and regular contact over time generate a richer understanding of needs and experiences [25] and TAR helped to situate discussions in specific consultations, thereby reducing recall bias. Longitudinal research, however, can make considerable demands on the respondents. Key to minimizing the impact of our research on patients was to keep the time-frame short (12 weeks), but sufficiently long to observe change. While single interviews offer an opportunity for respondents to reflect on their actions and make explicit their thought processes, they can also encourage accounts where respondents feel the need to justify actions and protect their self-presentation. Longitudinal research with regular patient contact enables interviewee-respondent relationships to form that can encourage greater openness about sensitive topics. This study therefore offers important insights into the effect of QOF on the organization of LTC management and patient care within review consultations in primary care.

Recordings of consultations can only provide a snap-shot and HCPs may argue that patients’ concerns have been addressed outside the recorded consultation. By speaking with respondents over time, and encouraging them to record healthcare contacts during that time, we were able to gain a fuller picture of people’s healthcare, their problems and their priorities, but it is still possible identified concerns could have been dealt with outside of the three-month follow-up period.

Research was conducted in a single PCT in North-West England with only six participating practices. We have no reason to believe that GPs and PNs in other parts of England organize care and offer review consultations in a different way to those in our study, but obviously extending this study to include more clinicians in other parts of England would be the logical next step. This would enable further investigation, in particular, of whether it is only GPs who feel able to deviate from the QOF review, or whether there are circumstances in which PNs are also able to respond to patient concerns and broaden the agenda to offer more patient-centred care under particular circumstances.

## Conclusions

Routine review consultations in primary care focus on the biomedical agenda set by QOF, where the practitioner is the expert. Moreover, they are shaping patients’ expectations and socializing patients into the role of passive subjects of ‘surveillance’.

The system in which practitioners work, determines activity in consultations. There is an obvious tension for practices because review consultations are needed to achieve QOF and thus maintain practice income. It is thus understandable that consultations focus on ‘ticking the boxes’. QOF does not reward demonstration of compassion/empathy, yet the rhetoric of primary care illustrated in the discourse of GPs and PNs in our study espouses a holistic, patient-centred approach. So, QOF prevails over the ethos of ‘holistic, patient-centred care’. However, there were practitioners and situations which prevailed over QOF – the prospect of death and bereavement [27] which allow HCPs the opportunity to offer truly patient-centred care.

The targets in QOF are evidence-based and therefore need to continue to be a part of care for patients with LTCs. However, there is a need for QOF to evolve [32] and to address the challenges posed by multimorbidity [33]. There is also a need to find ways of bringing the psycho-social domain and a patient-centred approach into consultations alongside, or as part of, QOF [34] in ways that do not reduce patient-centred care to ‘tick-boxes’. The inclusion of a ‘bio-psychosocial assessment’ for patients with newly diagnosed depression as a target in the revised QOF for 2013/4 [34] will challenge practices to deliver in any more than a further tick-box on the depression template.

Addressing the tensions between patient-centred care, responding to patient priorities versus delivering evidence-based care constrained and dictated by QOF might be achieved by longer consultation times [35], the use of innovative tools [36], and greater continuity [35] of care. HCPs need to be aware that patients have to do more than biomedical work to manage their LTC, and consideration is needed of how they can work with patients in these broader aspects of their lives, cognizant of multiple problems [2], and support and encourage patients to bring their concerns to the consultation.

## Abbreviations

(CHOICE): Choosing Health Options In Chronic care Emergencies; (GP): General Practitioner; (HCP): Healthcare Professional; (LTCs): Long-term conditions; (PN): Practice Nurse; (QOF): Quality and Outcomes Framework.

## Competing interests

The authors declare that they have no competing interests.

## Authors’ contributions

CCG designed the study, led the analysis of the data, and led the writing. CH conducted the research, analyzed the data, and contributed to the writing. SL contributed to the design of the study, contributed to the research, contributed to the analysis of the data, and contributed to the writing. AS conducted the research, contributed to the analysis of the data, and contributed to the writing. JD contributed to the analysis of the data, and contributed to the writing. EG designed the study, and contributed to the writing. PS designed the study, led the analysis of the data, and contributed to the writing. All authors have read and approved the final manuscript.

## Pre-publication history

The pre-publication history for this paper can be accessed here:

http://www.biomedcentral.com/1471-2296/14/103/prepub

## Supplementary Material

Additional file 1: Table S1 Numbers of audio-recordings and types of respondents. **Table S2**: Patients by practice, ID, age, gender, and condition/s. **Table S3**: HCPs by practice, ID, and role. Click here for file

Additional file 2 **Case 1.** Patient has informational needs–not met. Click here for file

Additional file 3 **Case 2.** Patient has emotional needs–not met. Click here for file

Additional file 4 **Case 3.** Patient has other biomedical needs–not met. Click here for file

Additional file 5 **Deviant case 1.** Shifting the priorities. Click here for file

Additional file 6 **Deviant case 2.** GP actively using the review to explore psychosocial needs. Click here for file
