# Supplementary material for: How QOF is shaping primary care review consultations: a longitudinal qualitative study
Source: BMC Fam Pract. 2013 Jul 21;14:103. doi: 10.1186/1471-2296-14-103 (PMC3726490; doi:10.1186/1471-2296-14-103)
Supplement: Additional file 1: Table S1 — Numbers of audio-recordings and types of respondents. Table S2: Patients by practice, ID, age, gender, and condition/s. Table S3: HCPs by practice, ID, and role. [file 1471-2296-14-103-S1.docx]

**Table S1** **Numbers of audio-recordings and types of respondents**

| Consultations | Patients | Patient Baseline Interview | Patient Follow up Interview | HCPs | HCP interviews |
| --- | --- | --- | --- | --- | --- |
| 32 | 28 | 28 | 23 | 10 | 10 |

**Table S2** **Patients by practice, ID, age, gender, and condition/s**

|  | **Practice** | **Patient ID** | **Age** | **Gender** | **Condition/s** |
| --- | --- | --- | --- | --- | --- |
|  | A | P01 | 70 | male | COPD, cancer |
|  | A | P02 | 62 | male | COPD, depression |
|  | A | P03 | 51 | female | Asthma |
|  | B | P02 | 46 | male | COPD |
|  | B | P04 | Not known | female | Diabetes, COPD |
|  | B | P05 | 85 | male | COPD, atrial fibrillation, dropped foot, balance problems |
|  | C | P01 | 51 | male | High blood pressure |
|  | C | P03 | Not known | male | Diabetes |
|  | C | P04 | 82 | male | Diabetes, Asthma |
|  | C | P05 | 54 | male | Diabetes, bowel problems |
|  | C | P07 | 47 | female | Diabetes, cancer |
|  | C | P09 | 87 | male | Heart disease, diabetes, depression |
|  | D | P02 | 65 | female | Diabetes, COPD, angina |
|  | D | P03 | 60 | male | Diabetes |
|  | D | P04 | Not known | female | CHD, Asthma |
| 1. P | D | P05 | 50 | female | Diabetes, nerve spasms |
|  | D | P06 | 76 | female | Heart disease, cancer, high cholesterol |
|  | D | P07 | 69 | female | COPD, arthritis |
|  | D | P08 | 74 | male | Heart disease, asthma, COPD, meningioma |
|  | D | P09 | 50 | male | Heart disease, depression, blindness |
|  | D | P11 | 43 | male | Diabetes |
|  | D | P13 | 62 | male | Heart disease, diabetes, high blood pressure, CKD |
|  | D | P14 | 58 | male | Heart disease, diabetes, cancer, piles |
|  | D | P15 | 57 | female | Asthma, sarcoidosis, bronchiectasis |
|  | D | P16 | Not known | female | Diabetes |
|  | E | P01 | 41 | female | Asthma |
|  | E | P02 | 51 | male | Asthma, high blood pressure |
|  | E | P03 | 73 | male | Heart disease, diabetes, gout |
|  | E | P07 | 30s | female | Asthma, depression, irritable bowel syndrome |
|  | E | P08 | 76 | male | Heart disease, high blood pressure, arthritis, asbestosis |
|  | E | P09 | 76 | female | Diabetes, arthritis |
|  | E | P13 | 67 | male | Diabetes, high blood pressure, glaucoma |
|  | F | P01 | Not known | male | Asthma, CHD |
|  | F | P03 | 67 | male | Heart disease, high blood pressure |

**Table 3** **HCPs by practice, ID, and role**

|  | Practice | HCP ID | HCP Role |
| --- | --- | --- | --- |
|  | A | HP06 | Practice Nurse |
|  | B | HP01 | Practice Nurse/Advanced Nurse Practitioner |
|  | C | HP02 | GP - Senior Partner |
|  | C | HP03 | GP Registrar |
|  | D | HP07 | Practice Nurse/Specialist Practitioner |
|  | D | HP09 | GP - Senior Partner |
|  | E | HP10 | Practice Nurse |
|  | E | HP11 | GP - Senior Partner |
|  | E | HP13 | GP Registrar |
|  | F | HP14 | Practice Nurse |
