# Supplementary material for: How QOF is shaping primary care review consultations: a longitudinal qualitative study
Source: BMC Fam Pract. 2013 Jul 21;14:103. doi: 10.1186/1471-2296-14-103 (PMC3726490; doi:10.1186/1471-2296-14-103)
Supplement: Additional file 2 — Case 1. Patient has informational needs–not met. [file 1471-2296-14-103-S2.docx]

***Case 1 Patient has informational needs – not met:***

***Practice A/P2***

Male, 62 years, white British, not working, lives with partner. Review appointment (COPD).

Patient presents with an ongoing problem of breathlessness when he exercises.

***Consultation***

*HP06/PN: So the only thing I can suggest really is to try this other clinic. You don’t want to start the road of nebulisers and, do you really?*

*A/P2: No, I don’t fancy that at all. I’m happy the way it is. I’m going to have my up and down days.*

HP06/PN seems to offer referral to a COPD clinic with little information about what this involves. The patient, without clarifying what a nebulizer is, and following the HP06/PN’s leading question, agreed that he would tolerate his symptoms.

***PN interview***

The above extract was played back to HP06/PN during the interview using TAR. HP06/PN was asked why she decided to defer referring patient to the COPD clinic:

*HP06/PN: I don’t think he needs a nebuliser (….) I know him quite well and he does tend to have burst of, where his symptoms seem to be worse and then they’ll be okay for a long while. So, yeah I’ll see him in a month and we’ll see how things are.*

Here, HP06/PN suggests that the patient does not need any further care, based on her familiarity with the patient, although the patient said that prior to this consultation, he had not been seen by the nurse for two years.

***Patient baseline interview***

During the interview, the patient expressed a sense of fatalism about his conditions.

*A/P2: I don’t think it would [help if the reviews were more regular], not, not, I think it’s just me, it’s just the way I am, I’ve always been that way, you know, it, if I feel alright carry on, if you don’t feel alright it’s time to go [*to the GP*], you know, I don’t think, I think if I went tomorrow [my illness]’d just be the same, or if I went in six month it would still be the same, you know.*

He disclosed concern that nebulisers would mean a loss of independence, confusing a nebulizer with oxygen therapy:

*A/P2: Well I think that if you start going on nebulisers you need to rely on them and I don’t, because I’ve seen one of my friends over there that needs oxygen and the way he’s actually ended up, and I don’t really want to end up like that.*

***Patient follow-up interview***

When asked about the value of review appointments, the patient said:

*A/P2: If I got a letter through the door and say, your review’s ready I’d go down, but apart from that I don’t really see that it’s necessary to go down (…) because as I say there’s not really a lot they can do, you know.*
