# Supplementary material for: How QOF is shaping primary care review consultations: a longitudinal qualitative study
Source: BMC Fam Pract. 2013 Jul 21;14:103. doi: 10.1186/1471-2296-14-103 (PMC3726490; doi:10.1186/1471-2296-14-103)
Supplement: Additional file 3 — Case 2. Patient has emotional needs–not met. [file 1471-2296-14-103-S3.docx]

***Case 2 Patient has emotional needs – not met:***

***Practice E/P1***

Female, 41 years, British South Asian, not working, separated. Review consultation (asthma).

Patient recently moved back to the UK following breakdown of her marriage. She has two young children.

***Consultation***

The patient had been invited to attend following an exacerbation of her asthma, which she, throughout the consultation, attributes to the stress of undergoing a divorce. On several occasions, HP10/PN disregarded the patient cues about stress, returning instead to the asthma template on the computer (in the example, below inhaler technique), as this example illustrates:

*E/P1: I'm so sorry [that I forgot to renew the inhaler], but like I say it's just not like me. I had, the horrible thing I'm thinking about is, I had a Court date on 12th December, and I think, I'm wondering now, it's not an excuse but...*

*HP10/PN: No.*

*E/P1: I lost track. Because I wrote it all down on my calendar, when to have my review is. So will I be able to get a prescription today, now?*

*HP10/PN: Yes, we'll give you a prescription today. I just want you to go through, have you got your inhaler? Do you carry it, your blue inhaler, with you?*

*E/P1: No.*

*HP10/PN: Okay. So you should always, I mean you know, we've said this at the last one, that is your rescue therapy, so you've found out the hard way haven't you really, of being […] out shopping, struggling to get your breath, it's very scary.*

***PN interview***

When asked to describe E/P1’s needs, HP10/PN responded:

*HP10/PN: She’s quite an anxious lady, sometimes she has her own agenda on some things, or they take priority and she doesn’t always take care of her, you know, the problem. She sees that problem as something that happens at the time and then goes away and if things improve she forgets all about it until the next time.*

When the nurse spoke about her role in medication use, it was in terms of encouraging adherence:

*HP10/PN: You’ve still got to get that message across that this is what you need to do and basically if you’re not complying and you’re not taking those inhalers well then the implications of that are you are going to get repeated exacerbations. You’re going to end up feeling horrible and panicking and like [E/P1] was, and that could be prevented as long as she complies with the medication.*

***Patient baseline interview***

The patient felt her concerns were dismissed by HP10/PN:

*E/P1: I’m really disappointed in [HP10/PN]. She’s very efficient and everything, but I didn’t feel that she had any empathy at all and the comment about, “Well you’ll remember for next time,” it’s just stayed with me.*

***Patient follow-up interview***

By the time of the follow-up interview, the patient was more positive about her relationship with the practice nurse, but has established limits on the types of problems she would raise with the practice nurse:

*E/P1: I am building more of a relationship [with HP10/PN], but again, if I'm brutally honest, I'm not that keen on seeing the nurse either. It's just because I don't really have any other option (…) it looks like we're getting back on track because I had really deteriorated with my asthma care. So, yeah, I'm literally, the way I see the surgery is it's for looking after my asthma, and it's the nurse [for my asthma care] and that's it.*
