# Supplementary material for: How QOF is shaping primary care review consultations: a longitudinal qualitative study
Source: BMC Fam Pract. 2013 Jul 21;14:103. doi: 10.1186/1471-2296-14-103 (PMC3726490; doi:10.1186/1471-2296-14-103)
Supplement: Additional file 4 — Case 3. Patient has other biomedical needs–not met. [file 1471-2296-14-103-S4.docx]

***Case 3 Patient has other biomedical needs – not met:***

***Practice C/P9***

Male, 87 years, retired, married. Review consultation (diabetes).

Has memory problems and urinary incontinence.

***Consultation***

HP02/GP opens the consultation with his agenda:

*HP02/GP: So there were two things really I need to discuss with you, one is your blood sugars, and we do a test which looks at the long-term control of your blood sugar, and it suggests that it probably could do with coming down a bit.*

*C/P9: Right, well...*

*HP02/GP: Now there are a couple of things you could do about that, one is the*

*alcohol.*

*C/P9: Knock it off?*

*HP02/GP: You could try and decrease it.*

*C/P9: Yeah.*

*HP02/GP: And the other is these new tablets that I'd like you to try.*

*C/P9: I'll try them.*

*HP02/GP: The other thing is, you could have a urine infection.*

*C/P9: A who?*

HP02/GP introduces the need for further blood tests as part of the QOF template for diabetes, and asks if the patient will also bring in urine samples. The GP twice brings up the need for patient to book these tests. Each time the patient at first appears to show he understands (‘right’, ‘ok’, ‘oh I see’) before going off on topics unrelated to the consultation. The patient seems overwhelmed by the requests:

*HP02/GP: Right, so you're going to do these two urine tests and drop them*

*back.*

*C/P9: I will.*

*HP02/GP: You're going to do, arrange the blood tests.*

*C/P9: Oh my god. This is very...*

*HP02/GP: I'll ring the front, I'll ring the front [reception] and tell them what you've got to do.*

HP02/GP recognizes that multiple instructions are causing confusion for the patient, but does not address this, other than taking control of the organization of the tests.

***GP interview***

HP02/GP argued that the key purpose of the chronic disease review appointments in the practice is to ensure that patients are viewed holistically by the GP who can ensure that care is coordinated:

*HP02/GP: One of the things we wanted to do when we designed our chronic disease review, was to have a slightly less specialised clinic, but one that dealt holistically with people with a range of problems (…) I would count us as the general physician who directs the care to the various places.*

***Patient baseline interview***

In contrast to the GP’s view of review appointments offering holistic care, the patient described having consultations with different doctors in the practice about different problems:

E/P9:  *There’s [HP02/GP], there’s [a second GP], and now I’ve got [a third GP, whom he saw for urinary incontinence]. Altogether, they can, they’ll probably pass it on, you know, “[The GP E/P9 [new to practice] saw for urinary incontinence], you’ve just arrived here, will you go and see this mithering brain [E/P9]”, you see’*

The patient describes himself as “this mithering brain”, a colloquial term meaning “bothersome” or “complaining”. He suspects that some doctors may pass him onto new doctors in the practice because he has so many ailments.

***Patient follow-up interview***

The patient and his wife described the number of different people involved in his care, and the confusion this caused:

*E/P9: Too many bloody pokers in the fire here.*

*Wife: We don’t know where we’re up to with [the care for his incontinence] really.*

In this instance, a referral had been made by another GP in the practice for him to see a specialist about his incontinence. Following this referral, the patient and his wife were given contradictory information about the appointment. They had asked for clarification at the GP practice, but said no-one had responded to them.
