# Supplementary material for: How QOF is shaping primary care review consultations: a longitudinal qualitative study
Source: BMC Fam Pract. 2013 Jul 21;14:103. doi: 10.1186/1471-2296-14-103 (PMC3726490; doi:10.1186/1471-2296-14-103)
Supplement: Additional file 5 — Deviant case 1. Shifting the priorities. [file 1471-2296-14-103-S5.docx]

***Deviant Case 1: shifting the priorities***

***Practice C/P7***

47 yrs old woman, white British, married with children. Review consultation (diabetes).

Has metastatic cancer (under specialist care)

***Consultation***HP03/GP gives the latest HbA1C result:

*HP03/GP: Then with your diabetes, the three month kind of average is [*pause*] what I’d call borderline.*

*C/P7: What is it?*

*HP03/GP: So it’s currently, (...) so it’s 61 which is 7.8, so*

*C/P7: Oh, that’s a little bit high (...) I don’t check my bloods I must be honest (…) But, I know this sounds awful but [*long pause - crying*] finding myself in this situation now, it’s kind of the least of my worries really [pause]. Sorry.*

*HP03/GP: You don’t need to apologise.*

*C/P7: You know I just think well, why worry about it, it’s something, it’s just really another thing, enjoy it while I’ve got some time left, you know.*

*HP03/GP: I think that’s really important (yeah) isn’t it, thinking about your priorities and thinking about, how we want to manage the full picture.*

The focus of consultation then moves to concerns raised by the patient around managing her pain and the need to know results of recent blood tests related to treatment for cancer, taken at the hospital. HP03/GP offered to obtain these results.

***GP interview***

The GP emphasized the importance of considering the ‘whole person’, even in a review consultation for a particular disease:

*HP03/GP: I think I remember from this kind of consultation one of the main skills that I try and use in this setting, in this chronic disease management setting, is really trying to understand from whoever’s in front of me, whichever patient’s come in, as to what they understand about the process and what they’re hoping to get out of coming along.*

***Patient baseline interview***

The patient had not expected HP03/GP to offer to find out blood tests that were unrelated to her ‘diabetes review’, but were concerning her:

*C/P7: So by him, offering to do that was quite brave but because maybe he’ll get an answer as to, should I be concerned or not about it.*

***Patient follow-up interview***

The patient was pleased that HP03/GP had obtained her blood test results relating to her cancer and shifted the emphasis from her diabetes, which was not her priority, and the knowledge that the GP and specialist knew each other seemed to have a positive impact upon her:

C/P7: *He gave me, he sent me a copy of the letter. The first part is to the GP’s surgery and then he’s put an additional note to dear [oncologist’s first name], that’s [name of] my oncologist because it turned out he knew him quite well, they trained together*.
