# Supplementary material for: How QOF is shaping primary care review consultations: a longitudinal qualitative study
Source: BMC Fam Pract. 2013 Jul 21;14:103. doi: 10.1186/1471-2296-14-103 (PMC3726490; doi:10.1186/1471-2296-14-103)
Supplement: Additional file 6 — Deviant case 2. GP actively using the review to explore psychosocial needs. [file 1471-2296-14-103-S6.docx]

***Deviant Case 2: GP actively using the review to explore psychosocial needs***

***Practice E/P8***

76 year old man, CHD, OA, asbestosis, white Irish, retired, widowed.

HP11/GP invited patient in for a ‘review’.

***Consultation***

HP11/GP opens the review by checking symptoms, medication and appointments for the patient’s cardiac problems. Once this is established, she moves on to explore his emotional state following his recent bereavement:

*HP11/GP: Okay that’s fine. So that’s in hand. And the chest pain’s a bit better, which is good.*

*E/P8: Yeah.*

*HP11/GP: Now how are you feeling in yourself because you were very low when we met last time?*

*E/P8: Yeah. It’s not got any better. I don’t know why. As I say I just can’t focus on things.*

In the rest of the consultation only 52 out of 244 lines of transcript are concerned with biomedical matters (medication, and specialist appointments) whilst the rest is about bereavement as a (physical and mental) health issue: loss of appetite, worries, lack of interest/s, feelings of isolation and anxiety, low mood. HP03/GP ends with asking him to book another appointment, and says:

*HP11/GP: You’ll get through this. We’ll get you through it*

***GP interview***

HP11/GP described how she uses the opportunity of a review consultation to support the patient:

*HP11/GP: I followed him up proactively, I asked him to come back and we booked that appointment mutually, so I wanted to follow him up because I was partly concerned to get his atrial fibrillation and his angina sorted, but also I knew that the death of his wife was going to have a massive impact on him so I wanted t*o *follow that up.*

She described her views of QOF:

*HP11/GP: I think one of the dangers with QOF is it’s very reductionist. There’s nothing in QOF that says have you ever asked a bereaved man how crap he feels because his wife’s died. That’s not in the QOF anywhere. And yet in terms of looking after that patient, I think that consultation was a very significant one. It’s what you would want from your doctor isn’t it? Is somebody to pay attention to you as a human being in your real actual situation? If I just said to him, “Well, up your Isosorbide and reduce your aspirin by” in what way would that be helping? I don’t think it would you see (…) Now all doctors do those other aspects but it isn’t incentivised and it isn’t valued by the health service at all. That consultation with that guy that probably stopped him killing himself isn’t valued at all.’*

HP11/GP defined the role of the GP as more than the management of disease:

*HP11/GP: I talk to people about bereavement and death all the time. General practice is a meditation on the theme of loss, that’s what we do. People have lost their health… that conversation was about loss and its aftermath, and what you do and how you cope. So it’s about being with people in their suffering isn’t it?*

***Patient baseline interview***

The patient described how he valued the opportunity to consult HP11/GP about his bereavement:

*E/P8: You have just got to have faith in [HP11/GP] and that she will do the right thing for you, and look after you. Which she has been doing (…) And I am going to see her tomorrow afternoon, and she will be asking me how I am doing with the problem with [the death of his wife] now. Because I did go through a really bad patch, and she says it will get better hopefully.*

No follow-up interview was conducted with this patient as he was upset in the initial interview, and it was felt to be inappropriate to conduct the three month follow-up.
